# Do Probiotics Improve the Health Status of Individuals with Diabetes Mellitus? A Review on Outcomes of Clinical Trials

**DOI:** 10.1155/2019/1531567

**Published:** 2019-12-14

**Authors:** Periyanaina Kesika, Bhagavathi Sundaram Sivamaruthi, Chaiyavat Chaiyasut

**Affiliations:** Innovation Center for Holistic Health, Nutraceuticals, and Cosmeceuticals, Faculty of Pharmacy, Chiang Mai University, Chiang Mai 50200, Thailand

## Abstract

Probiotics are now considered as an adjuvant and complementary therapeutic agent for several health complications, especially for metabolic and gastrointestinal disorders because of the influential impact of probiotic consumption on gut microbiota and immunity. Diabetes mellitus (DM) is fourth, in noncommunicable disease category, leading cause of mortality, morbidity, and economic crises in the world. Though several progressions are added in the medical field in recent decades, the treatment and management of diabetic-related health issues are still challenging. The present study summarizes the effects of probiotic supplementation on the health status of diabetic patients. The relevant information was collected from Scopus, PubMed, and Google Scholar. The detailed literature survey revealed that the consumption of probiotic supplementation significantly improved the overall health condition of diabetic patients. Especially, the probiotic intervention improved the fasting blood glucose, insulin sensitivity, and systemic inflammatory and antioxidant status in type 2 diabetic (T2D) patients. Moreover, improvement of gut microbial composition and prevention of bacterial translocation has also been observed in probiotic-supplemented T2D people. Some of the studies evidenced that the supplementation of probiotics can prevent and improve the gestational DM. Nevertheless, some of the studies reported negative results and limitations in the results of clinical trials. However, further studies are mandatory to develop a concrete probiotic-based adjuvant treatment procedure to treat DM.

## 1. Introduction

Diabetes mellitus (DM) is one of the major threats to human health and considered as the fourth most common noncommunicable diseases (NCD). A recent report in Lancet Global Health [1] revealed that diabetes-associated health loss increased drastically in India since 1990, which is highest among the NCD. The incidence of DM in the US also increasing at an uncontrolled rate, and the number of Americans with DM might increase up to 55 million by 2030 [2]. The global costs of management of diabetes and its consequences are increasing and predicted that it may reach up to US$2.1 trillion in 2030 [3].

Type 2 DM (T2D) accounts for 90–95% of DM incidences and is characterized by high blood glucose level, insulin deficiency and resistance [4]. Untreated DM is associated with the development of health difficulties that may compromise the quality of life and may increase the risk of mortality. T2D also affects the psychological and social life of most of the patients, and T2D patients are often found with some psychological problems like stress, depression, and anxiety [5].

Apart from heredity, dietary behavior, lack of physical activities, obesity, and some medication are the major factors associated with the incidence of DM. Recent studies evidenced that host microbiome play a critical role in the incidence and management of DM, especially in T2D [6]. The inconsistency of gut microbiota (GM), termed as dysbiosis, is greatly associated with T2D, and it has been reported that GM of T2D patient comprised of opportunistic pathogens and reduced number of beneficial microbes, especially butyrate-producing bacteria [7]. The improvement of gut health may aid to manage the diabetic conditions that may prevent the development of further DM-associated health complications.

Probiotics are live microbes that, when consumed in sufficient amounts, confer a health benefit to the humans [8]. Probiotics have been recognized for their several health-promoting properties, and ameliorating the physiological and psychological hitches and distress [9–13]. Though several studies reported that *Lactobacillus* species confer positive impact on host health, the beneficial effects of probiotics are strain-specific, and also related to the host physiology. Recently, *Akkermansia muciniphila *has been considered as a potential bacteria that could be used to treat T2D, and *A. muciniphila* improves the glucose intolerance in diabetic mice [14], but the use of *A. muciniphila *in human need further advancement in the preparation of the therapeutic agent and detailed validation.

**Figure 1:**
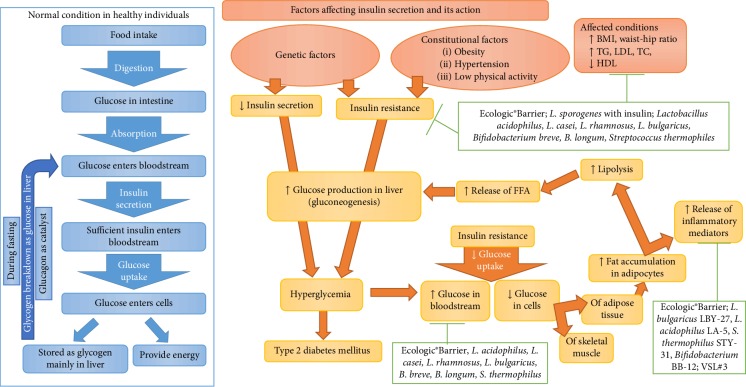
Probiotics targeting the cellular and molecular mechanism of type 2 diabetes mellitus (T2DM). The left part of figure represents the normal glucose metabolism and regulation that occurs in a healthy nondiabetic individual (when a human ingest food, it gets digested and carbohydrate breakdowns into glucose in the intestine. Then glucose enters the bloodstream. The *β* cells of pancreas releases sufficient amount of insulin into the bloodstream inresponse to the glucose content. Glucose uptake by cells of muscle and adipose tissues are promoted by the insulin. The endogenous insulin secretion suppresses the secretion of glucagon in pancreas. The unused remaining glucose are stored as glycogen mainly in liver. Glycogenolysis and gluconeogenesis in liver is suppressed by the insulin. And finally, the blood glucose level reaches the normal level. When blood glucose concentration is reduced than a normal level during fasting, the *α* cells of pancreas secretes glucagon and releases glucagon in to liver. Glucagon catalyses the conversion of glycogen into glucose and liver releases the glucose in to the bloodstream. Glucose uptake by cells of muscle and adipose tissues are controlled by the basal level of insulin and the blood glucose level is maintained. Glycogenolysis and gluconeogenesis in the liver is minimally suppressed due to to the low level of insulin secretion). The right part of the figure represents the impared glucose metabolism and regulation in individuals with T2DM (genetic and constitutional factors affect the secretion and action of insulin. *β* cell dysfunction in diabetic (T2DM) individual is caused due to the effect of FFAs, insulin resistance, obesity, and inflammation. The *β* cell function is affected by long term exposure of FFAs. Initially, the short term exposure of FFAs after ingestion of mixed meal causes an increase in the level of insulin secretion and allows the storage of extra calories as fat, which eventually results in overweight and leads to obesity. It also accounts for the increased secretion of insulin in response to insulin resistance. But glucose induced insulin secretion is suppressed due to the long term exposure of FFAs. Insulin resistance in obese individual causes increased demand on *β* cell function that leads to metabolic exhaustion of *β* cells and accelerates the loss of *β* cell mass. Incase of *β* cell dysfunction, the insulin secretion is reduced due to loss of *β* cell mass. The low level of endogenous insulin secretion does not effectively suppress the rate of glucagon secretion in pancreas. Resulting in increased glycogenolysis and gluconeogenesis in liver and releasing more glucose in bloodstream, which occurs both at fasting and fed state. Incase of insulin resistance, the insulin mediated uptake of glucose is reduced in skeletal muscles and adipose tissues that results in increased blood glucose level following the ingestion of food. The upregulated accumulation of fat in the cells of adipose tissues increases the release of pro-inflammatory mediators and causes increased lipolysis, which results in releasing more FFAs that induces glucose production in liver and leads to progressive hyperglycemia) and probiotics targeting the mechanism of T2DM to improve the health status of individuals with T2DM (probiotics improves the glycemic status by reducing the blood glucose level, insulin resistance, the production of inflammatory markers and increasing fasting insulin level, improvement of lipid profile by increasing LDL and decreasing TC, TG, HDL, and improvement of hypertension by decreasing the systolic and diastolic blood pressure in individuals with T2DM). The orange lines indicate the mechanism involved in T2DM. Green lines indicate the targets of probiotic. T2DM: Type 2 diabetic mellitus; Ecologic®Barrier is a probiotic mixture of *Bifidobacterium bifidum* W23, *B. lactis* W52, *Lactobacillus acidophilus* W37, *L. brevis* W63, *L. casei* W56, *L. salivarius* W24, *L. lactis* W19, and *Lactococcus lactis* W58; VSL#3 is a probiotic mixture of *L. acidophilus, L. plantarum, L. paracasei, L. delbrueckii* subsp. *Bulgaricus, B. breve, B. longum, B. infantis*, and *S. thermophiles*; BMI: Body mass index; TG: Triglycerides; LDL: Low-density lipoprotein; HDL: High-density lipoprotein; TC: Total cholesterol; FFA: Free fatty acid.

In recent decades, several clinical trials have been conducted to evaluate the antidiabetic nature of probiotic preparations. All the studies have their merits and demerits, and the results are inconclusive. Numerous meta-analysis reports also emphasized the key findings of the clinical trials and specified some points that need to be considered in clinical trials. The current manuscript was prepared with the aim of summarizing the significant findings of antidiabetic properties of probiotics concerning the outcomes of studies conducted in human volunteers. The antidiabetic activity of the probiotics on the mechanism of T2DM conditions are illustrated in Figure 1.

## 2. Antidiabetic Properties of Probiotic Supplements

### 2.1. Probiotic Supplement

The supplementation of a mixture of probiotics strains such as *Bifidobacterium bifidum* W23, *B. lactis* W51, *B. lactis* W52, *L. acidophilus* W37, *L. brevis* W63, *L. casei* W56, *L. salivarius* W24, *Lactococcus lactis* W19, and *Lactococcus lactis* W58 (Ecologic®Barrier) at the final concentration of 2.5 billion cells per gram (two gram per day) for 26 weeks were expected to significantly reduce the systemic inflammatory status, inflammatory responses, endotoxins level in T2D patients [15]. Though Alokail et al. [15] already reported a study protocol to investigate the beneficial impact of Ecologic®Barrier in T2D patients, a recent study [16] followed the study protocol [15] and showed that the short term (12 weeks) supplementation of Ecologic®Barrier improved the HOMA-IR value and some anthropometric parameters (waist-hip ratio), but no changes were observed in the endotoxin level in T2D patients [16]. Sabico et al. [17] extended the study following the protocol [15] with extended duration (6 months) to evaluate the potential of Ecologic®Barrier. The results revealed that the supplementation of Ecologic®Barrier significantly reduced the circulating endotoxin level, fasting plasma glucose (FPG), TG (triglycerides), HOMA-IR (Homeostasis model of assessment-insulin resistance), insulin, total cholesterol (TC), TC: HDL (high-density lipoprotein) ratio, inflammatory markers (TNF-*α*, IL-6), C-reactive protein (CRP), resistin in T2D patients. Moreover, the increase in adiponectin concentration was observed after probiotic supplementation. The results revealed that the supplementation of probiotics may improve the health status of T2D patients if the intervention lost for an optimum duration [17].

The intervention of probiotic preparation consists of a various concentration of *L. acidophilus*,* L. casei*, *L. rhamnosus*,* L. bulgaricus*,* B. breve*, *B. longum, *and *Streptococcus thermophilus *strains along with fructo-oligosaccharide (FOS) for 8 weeks significantly reduced the fasting plasma glucose (FPG) level in T2D patients compared to that of the placebo control. The level of both serum insulin and LDL (low-density lipoprotein) was increased in the probiotic and placebo group. HOMA-IR score was increased compared to baseline but HOMA-IR score was relatively low compared to that of the placebo control. Moreover, probiotic supplementation significantly improved the level of glutathione in T2D patients, which suggested that probiotic supplementation improved the antioxidant status of the patient and control the diabetic condition by reducing the glucose level [18]. Likely, the supplementation of multispecies probiotic preparation along with 100 mg FOS significantly increased the serum calcium level and decreased the ALT (alanine aminotransferase) in T2D patients, while no influence on other serum minerals such as zinc, iron, magnesium, and liver enzymes such as alkaline phosphatase and aspartate aminotransferase were observed [19]. Recently, a study reported that the same multistrain probiotic intervention along with 100 mg FOS improved the glycemic status of T2D patients. Even though the notable amount of reduction was observed in FPG and increase in HDL, there were no changes in insulin resistance, TG, TC and insulin levels, and anthropometric values like BMI (body mass index), chest, hip, and waist circumferences. Yet supplementation of multistrain probiotic preparation with FOS improved the FPG status in T2D patients, further studies are needed to confirm the beneficial effect of the probiotic interventions [20].

The supplementation of synbiotic preparation (1 × 10^7^ CFU of *L. sporogenes*, 40 mg inulin, 380 mg isomalt, and 360 mg sorbitol, and 50 mg stevia per gram; 9 g of synbiotic thrice a day) for six weeks significantly improved the metabolic status of T2D patients. Specifically, the synbiotic group showed a notable level of decrease in serum insulin, hs-CRP levels, and increased glutathione and uric acid level compared to control. There were no significant changes noticed in TC value, HOMA-IR score, and LDL level. Also, the total antioxidant capacity of the system was not affected by the synbiotic intervention. The results showed that synbiotic supplementation can improve the metabolic status of T2D patients [21].

The intervention of multistrain probiotic mixture (10^10^ CFU per day; *L. acidophilus, L. casei, L. lactis, B. bifidum, B. longum,* and* B. infantis*) for 12 weeks showed no significant improvement in HOMA-IR, QUICKI (Quantitative insulin sensitivity check index), hs-CRP, and lipid profile except modest improvement of fasting insulin and HbA1c levels [22].

The consumption of *L. reuteri *ADR-1 and *L. reuteri* ADR-3 improved the fecal microbial composition and DM-associated complications in T2D patients. The consumption of live cells of probiotic confers better health benefits compared to that of the heat-killed cells. The level of HbA1c, TC and blood pressure was found to be decreased significantly. The fecal *Lactobacillus* and *Bifidobacterium* load were also increased after the probiotic intervention, which was positively correlated with the amount of *L. reuteri *in the fecal sample. The antioxidant and inflammatory status of the patients were not affected by the probiotic supplementation. Both the strains of *L. reuteri* exhibited health-promoting property with unique influences on fecal microbiota and other DM-associated parameters [23].

T2D patients were supplemented with two different doses (10^8^ or 10^10 ^CFU per day) of a single strain of *L. reuteri* DSM 17938 for 12 weeks and changes in the selected biomarkers were assessed. The results showed that DSM 17938 consumption did not improve the HbA1c, and biomarkers related to adiposity, liver steatosis, and fecal microbial composition, whereas, consumption of a high dose of DSM 17938 improved the insulin sensitivity index and serum deoxycholic acid, which was correlated with each other and microbial composition. The study claimed that further studies are required to confirm the findings with respect to individual variations [24].

The supplementation of Symbiter (a combination of different concentrations of 14 probiotic strains of *Bifidobacterium, Lactobacillus, Lactococcus* and *Propionibacterium* species, and *Acetobacter* genera) for eight weeks improved the insulin resistance in T2D patients. Specifically, the HOMA-IR score and HbA1c value were found to be reduced significantly in the probiotic-supplemented group, especially among the probiotic responders, compared to that of the placebo, but no significant changes were observed in inflammatory markers such as IL-6, IL-8, and INF-*γ* except TNF-α and IL-1β [25].

T2D patients were either supplemented with synbiotic formula (500 mg/day; containing *Lactobacillus*, *Bifidobacterium* species, *S. thermophilus,* and FOS) or placebo formula for nine weeks. The influence of synbiotic supplementation on DM-associated parameters has been assessed. The results showed that the test intervention significantly reduced the FPG, HbA1c, and BMI values compared to that of the control group, whereas, there was no significant improvement in lipid, TC, urea, and creatinine profile in both groups at the end of the study. The study concluded that the supplementation of a specific amount of specified synbiotic formulation merely improved the FPG, HbA1c, and BMI value in T2D patients [26].

The consumption of synbiotic shake containing *L. acidophilus, B. bifidum, *and FOS for thirty days significantly improved the glycemic status and reduced the TC and TG levels in T2D patients. A notable level of improvement in HDL content was also observed in synbiotic shake supplemented group compared to that of the placebo [27].

The supplementation of synbiotic supplements (2 × 10^9^ CFU of each *L. acidophilus, L. casei *and *B. bifidum*, and 800 mg inulin per day) for 12 weeks significantly reduced the FPG, insulin, and HOMA-*β* cell function score, and increased the insulin sensitivity index and HDL content in obese T2D patients with coronary heart disease compared to that of the placebo control and baseline values [28].

The supplementation of the probiotic mixture (*L. salivarius* UBLS 22, *L. casei* UBLC42, *L. plantarum *UBLP40, *L. acidophilus* UBLA34, *B. breve* UBBR 01, *Bacillus coagulans *Unique‑IS2) and prebiotic (FOS) for 12 weeks improved the glycemic control and health status of T2D patients compared to that of the placebo control. Based on the intragroup analysis, the study stated that the health benefits of synbiotic supplementation correlated with the age of the patients while not associated with gender variation [29].

Gestational DM (GDM) is one of the metabolic complications of pregnancy to the susceptible individuals with the incidence of 3–25%, and probiotic supplementation may ameliorate glycemic control in GDM patients [30]. The supplementation of the probiotic capsule that consists of four probiotic strains (*L. acidophilus* LA-5, *L. delbrueckii bulgaricus* LBY-27, *S. thermophilus* STY-31, and *Bifidobacterium* BB-12; 4 × 10^9^ CFU in total) for eight weeks hindered the weight gaining process after 6 weeks of the intervention compared to the placebo group in recently diagnosed GDM patients. The level of FPG was reduced in all the subjects, but significant reduction was observed in the probiotic-supplemented group. Insulin resistance index and Insulin sensitive index (ISI) were reduced and increased in the probiotic group, respectively, but the ISI value was not significantly different from that in the placebo group [31]. Similarly, the antioxidant status and inflammatory profile of the GDM patients were found to be improved after the probiotic (*L. bulgaricus* LBY-27, *L. acidophilus* LA-5, *S. thermophiles* STY-31, *Bifidobacterium* BB-12) intervention [32]. The results suggested that probiotic intervention has a beneficial impact on GDM patients and a further detailed study is needed to claim the therapeutic credit to the studied probiotic preparation.

About eight weeks of supplementation of VSL#3 (a mixture of *L. acidophilus, L. plantarum, L. paracasei, L. delbrueckii* subsp. *Bulgaricus, B. breve, B. longum, B. infantis*, and *S. thermophilus*; 1.125 × 10^11^ CFU per capsule) failed to improve the FPG and HbA1c values in GDM patients, whereas, a significant level of reduction was observed in IL-6, hs-CRP, TNF-*α* concentration after probiotic supplementation. HOMA-IR value and insulin levels were also different among the probiotic and control groups while no notable changes were observed in IL-10. Further studies are required to conclude the beneficial effect of VSL#3 on the health status of GDM patients [33]. Interestingly, supplementation of the probiotic mixture (*L. acidophilus*, *L*. *casei*, and *B. bifidum*; 2 × 10^9^ CFU each) significantly amended the health status of GDM patient. A significant reduction of FPG, TG, VLDL (very low density lipoprotein), insulin level, HOMA-IR, and HOMA-*β*-cell function were observed in the probiotic supplemented group compared to that of the placebo control after 6 weeks. The insulin sensitivity of the patient was also increased at a notable level, while the lipid profile was not improved during the study period. The study claimed that the supplementation of prescribed probiotic formula at a specified concentration may improve the overall health status of GDM patient [34]. A four-week supplementation of Infloran® (10^9^cells each; *L. acidophilus*, and *B. bifidum*) to GDM patients improved the metabolic status of the subjects. A notable level of improvement was observed in FPG, fasting insulin level, and HOMA-IR score in the probiotic-supplemented group compared to that of the placebo control. There was no change in weight gain between the groups. The results disclosed that the supplementation of probiotic could improve glycemic control in GDM patients [35]. The supplementation of probiotic strain *L. rhamnosus* HN001 to pregnant women, starting from 14 to 16 weeks of gestation, reduced the risk of development of GDM, especially in older women with the previous history of GDM [36].

DM preventing properties of probiotic supplementation has also been studied in pre-diabetic people. Effect of supplementation of probiotic or synbiotic preparation on glycemic control of pre-diabetic people has been studied in a double-blind, randomized controlled clinical trial. The subjects were randomly divided and supplemented with probiotic (each 10^9^ CFU; *L. acidophilus, B. lactis, B. bifidum, *and* B. longum*) or synbiotic (probiotic + inulin) or placebo for 24 weeks. The analyses of selected parameters showed that the supplementation of synbiotic preparation significantly reduced the FPG, HbA1c, fasting insulin level, and HOMA-IR score, and also increased the QUICKI score compared to that of the placebo control and probiotic group. HOMA-*β*-cell function score was found to be not affected by any of the intervention. The results of the study suggested that the intervention of synbiotic preparation may reduce the risk of developing the metabolic disorder in prediabetic people, but detailed studies are needed [37]. In another study, Kassaian et al. [38] studied the beneficial effect of probiotic and synbiotic formulation, slightly increased concentration (each 1.5 × 10^9^ CFU), in pre-diabetic people. The results showed that the supplementation of probiotic and synbiotic may reduce the risk of metabolic syndrome, and also aids to manage the metabolic disorders [38] (Table 1).

### 2.2. Probiotic Soymilk

The supplementation of probiotic soymilk improved the kidney function in T2D patients. Eight-week randomized-controlled study with T2D patients showed the beneficial impact of consumption of soymilk containing *L. plantarum *A7 (200 ml per day) in terms of improvement of kidney function. The studied parameters such as urine albumin level, serum creatinine, sialic acid, and IL-18 levels were reduced in the probiotic-supplemented group compared to that of the control, and a significant level of improvement in estimated glomerular filtration rate was also observed in the treatment group. The results suggested that the supplementation of *L. plantarum *A7 containing soymilk improved the kidney function [39]. Similarly, the supplementation of probiotic soymilk improved some of the oxidative stress markers in patients with diabetic kidney disease (DKD). Specifically, about eight-week consumption of probiotic soymilk increased the level of reduced glutathione, glutathione peroxidase, and glutathione reductase in DKD patients, while no changes were observed in MDA (malondialdehyde) and 8-iso-prostaglandin F2*α* values, and total antioxidant capacity [40].

### 2.3. Fermented Milk Containing Probiotic

The consumption of 300 g of probiotic yogurt (PY; comprising of *L. acidophilus* La5 and *B. lactis* Bb12) for six weeks significantly improved the lipid profile in T2D patients. The level of TC and LDL was reduced in PY supplemented group compared to baseline and to that of the conventional yogurt group (control). Though no significant changes were observed in TG and HDL, the ratio of TC: HDL, and LDL: HDL was reduced in the probiotic group [41]. In another arm, the consumption of PY improved the antioxidant status of T2D patients. The level of SOD (superoxide dismutase), GPx (glutathione peroxidase), and TAS (total antioxidant status) was increased in PY group compared to that of the control and baseline value. The level of MDA was reduced after PY consumption, while no significant changes were observed in insulin and CAT (catalase) levels [42]. Overall, results suggested that the consumption of probiotic yogurt may reduce the risk of cardiovascular diseases and improved the radical scavenging system in T2D patients [41, 42]. Another study by Rezaei et al. [43] showed that consumption of 300 g of PY per day for four weeks significantly improved the glycemic status (reduced the FPG, and HbA1c), and serum lipid concentration (reduced the LDL) compared to baseline values [43]. Drinking fermented milk containing *L. casei *strain Shirota (FMLCS) for 16 weeks significantly altered the fecal microbiota, and organic acid content. Specifically, the probiotic intervention increased the population of *Lactobacillus *species, *Clostridium coccoides *group and *C. leptum *subgroup in the fecal microbial community. The number of blood bacteria was significantly reduced after the consumption of FMLCS and also hindered the bacterial translocation in T2D patients [44]. Similarly, the consumption of fermented milk containing *L. acidophilus* La-5 and *B. animalis* subsp. *lactis* BB-12 for 6 weeks showed significant changes in HbA1c, fructosamine levels, and reduced the inflammatory marker (TNF-*α*), resistin in T2D patients. The lipid profile of the probiotic-supplemented group was found to be improved (reduced the TC, and LDL content). The fecal organic acid assessment showed that fermented milk consumption increased the acetic acid content in fecal samples of the participants. The results suggested that probiotic consumption improved the glycemic control and inflammatory status of T2D patients [45].

Most of the studies evaluated the health-promoting effect of *Lactobacillus*, *Bifidobacterium* and *Streptococcus* species, especially *L. acidophilus*, *B. breve, *and *S. thermophilus*, respectively, in DM patients. The health-promoting potential of the intervention was found to be varied based on the type of the strain, concentration, and combinations of other probiotic strains, and also cointervention of prebiotics.

## 3. Probiotic Supplementation Showing No Positive Effect on Diabetic Condition

Though several studies showcased the potential of probiotic supplementation in the improvement of the health status of DM patients, some of the studies displayed negative results. The consumption of single strain probiotic (*L. salivarius* UCC118) for six weeks had no significant impact on the metabolic and glycemic status of GDM patients [46]. The supplementation of synbiotic formula (*L. casei*, *L. acidophilus*, *L. rhamnosus*, *L. bulgaricus*, *B. breve*, *B. longum*, *S. thermophilus *+ FOS) showed no beneficial effect on the lipid profile (TC, LDL, HDL, TG) in prediabetic patients [47]. Mazloom et al. [48] also reported the nonsignificant changes in the glycemic status of T2D patients after the consumption of probiotic preparation (*L. acidophilus*, *L. bulgaricus*,* L. casei*, and *B. bifidum*) for six weeks.

## 4. Concluding Remarks

Several meta-analysis studies on results of clinical trials suggested that probiotic supplementation reduced the FPG, lipid profile, blood pressure, and other cardiovascular risk factors in T2D patients [49–53]. The probiotic supplements effectively controlled the glycemic and inflammatory status of GDM patients [30]. The possible mechanism of probiotics include increasing the fasting insulin concentration to reduce the FPG, increasing HDL and decreasing TC, TG, LDL to improve the lipid profile, decreasing the systolic and diastolic blood pressure to maintain the normal blood pressure. Thereby, probiotics reduces cardiovascular risk by controlling hypertension and dyslipidemia in individuals with T2DM. Probiotics controls the glycemic condition by reducing the insulin resistance and might decrease the production of inflammatory markers in individuals with T2DM. Probiotic formulations showed promising results in most of the studies, except in some cases [46–48].

In general, clinical studies were conducted in a prescribed procedure. Though most of them are randomized, blinded, placebo-controlled studies, some of the limitations are there in any one or more of the following: sample size, preparation of supplements, monitoring of intervention, sampling, inconsistency in measurements, bios in the questionnaire, and rarely, study protocol. In addition, patient's physiology, combination and types of probiotics used, and experimental duration and follow-up period also influence the outcome of clinical trials. Thus, further well-designed studies are needed to understand the link between the role of the microbiome, the interaction of probiotics with host microbiota, and glycemic control.

## Figures and Tables

**Table 1:** The anti-diabetic properties of probiotics supplementations: outcomes of randomized, double-blind, controlled clinical trials using human subjects.

Subjects	Intervention	Duration	Key observations	Health claim	Reference(s)
T2D patients	Soy milk with *L. plantarum *A7	8 weeks	↓ Urine albumin level	Improved the kidney function	[39]
			↓ Serum creatinine, IL-18, sialic acid levels Improved the GFR		
DKD patients	Probiotic soy milk	8 weeks	↑ Reduced GSH, GPx, glutathione reductase	Improved the oxidative stress factors	[40]
T2D patients	Ecologic®Barrier	26 weeks	↓ Systemic inflammatory state, and inflammatory response	Improved the systemic inflammatory status	[15]
			↓ Endotoxin levels altered the gut microbiota		
T2D patients	Ecologic®Barrier	12 weeks	↓ Waist-hip ratio Improved HOMA-IR	Improved HOMA-IR score	[16]
T2D patients	Ecologic®Barrier	6 months	↓ Endotoxin	Improved HOMA-IR score, and cardiometabolic profile	[17]
			↓ TG, TC, HOMA-IR		
			↓ TNF-α, IL-6		
			↓ C-reactive protein, resistin		
			↑ Adiponectin		
T2D patients	*L. acidophilus L. casei, L. rhamnosus, L. bulgaricus, B. breve, B. longum, *and *Streptococcus thermophilus*	8 weeks	↓ Fasting plasma glucose (FPG)	Improved the antioxidant status and mineral content	[18, 19]
			↑ Serum insulin, LDL level, HOMA-IR score Altered the hs-CRP value		
			↑ Serum GSH level		
			↑ Serum calcium level		
			↓ Serum ALT		
T2D patients	*L. acidophilus, L. casei, L. rhamnosus, L. bulgaricus, B. breve, B. longum, S. thermophilus *and FOS	6 weeks	↓ FPG	Improved the glycemic status	[20]
			↑ HDL No changes in TG, TC, insulin level, anthropometric values		
T2D patients	*L. sporogenes *and inulin	6 weeks	↓ Serum insulin level, FPG, hs-CRP levels	Improved the metabolic status	[21]
			↑ Serum GSH level, uric acid		
T2D patients	Yogurt containing *L. acidophilus* La5 and *B. lactis* Bb12	6 weeks	↓ TC, LDL	Improved the lipid profile and TAS	[41, 42]
			↓ TC: HDL, and LDL: HDL ratio		
			↓ FPG, HbA1c, MDA		
			↑ SOD, GPx, and TAS		

T2D patients	Yogurt containing *L. acidophilus* La5 and *B. lactis* Bb12	4 weeks	↓ FPG, HbA1c, TG	Improved the lipid profile and glycemic status	[43]
			↓ LDL		
T2D patients	*L. acidophilus, L. casei, L. lactis, B. bifidum, B. longum,* and* B. infantis*	12 weeks	↓ HbA1c, Fasting insulin level	Improved only HbA1c, fasting insulin level	[22]
T2D patients	*L. reuteri *ADR-1, and* L. reuteri* ADR-3	9 months	↓ HbA1c, Cholesterol level	Improved the fecal microbial composition	[23]
			↓ Blood pressure		
			↑ Fecal *Lactobacillus* and *Bifidobacterium* load		
T2D patients	*L. reuteri* DSM 17938	12 weeks	↑ Insulin sensitivity index	Improved insulin sensitivity	[24]
			↑ Serum deoxycholic acid		
T2D patients	Symbiter	8 weeks	↓ HOMA-IR, HbA1c	Improved the insulin resistance	[25]
T2D patients	*L. casei *strain Shirota fermented milk	16 weeks	↓ Bacterial translocation	Reduced the bacterial translocation	[44]
			↑ *Clostridium coccoides *group		
			↑ *C. leptum *subgroup, *Lactobacillus *species		
T2D patients	Fermented milk (*L. acidophilus* La-5 and *B. animalis* subsp. lactis BB-12)	6 weeks	↓ HbA1c, fructosamine levels	Improved the glycemic control	[45]
			↓ TNF-*α* and resistin		
			↓ TC, LDL		
			↑ Fecal acetic acid		
T2D patients	*Lactobacillu*s spp., *Bifidobacterium* spp., *S. thermophilus*, and FOS	9 weeks	↓ FPG, HbA1c, BMI	Improved the glucose level and DM-associated parameters	[26]
			↓ Microalbuminuria		
T2D patients	*L. acidophilus, B. bifidum *and FOS	30 days	↓ FPG, TC, TG, LDL	Improved the glycemic status	[27]
			↑ HDL		
T2D patients with CHD	*L. acidophilus, L. casei, B. bifidum* and inulin	12 weeks	↓ FPG, insulin level, HOMA-*β* cell function	Improved the insulin metabolism	[28]
			↑ Insulin sensitivity index, HDL		
T2D patients	*L. salivarius *UBLS, *L. casei *UBLC42, *L. plantarum *UBLP40, *L. acidophilus *UBLA34, *B. breve *UBBR01, *Bacillus coagulans *Unique‑IS2, and FOS	12 weeks	↓ FPG, postprandial blood sugar, insulin level, HOMA-IR	Improved the HRQL	[29]
GDM patients	*L. acidophilus* LA-5, *Bifidobacterium *BB-12, *S. thermophilus* STY-31, and* L. delbrueckii bulgaricus *LBY-27	8 weeks	↓ Weight gain	Influenced the weight gain and glucose metabolism	[31]
			↓ FPG		
			↓ Insulin resistance index		
			↑ Insulin sensitivity index		
GDM patients	*L. acidophilus* LA-5, *Bifidobacterium *BB-12, *S. thermophilus* STY-31, and* L. delbrueckii bulgaricus *LBY-27	8 weeks	↓ TNF-*α*	Improved inflammation and antioxidant status	[32]
			↓ hs-CRP value		
			↓ IL-6, MDA, uric acid		
			↑ Glutathione reductase, SOD, TAS		
GDM patients	VSL#3	8 weeks	No change in FPG, HbA1c values, and insulin level	Improved the inflammatory markers	[33]
			↓ IL-6, hs-CRP, TNF-*α*		
GDM patients	*L. acidophilusL*. *casei *and *B. bifidum*	6 weeks	↓ FPG, insulin level, HOMA-IR, HOMA-*β*-cell function	Glycemic control	[34]
			↓ TG, VLDL		
			↑ Insulin sensitivity		
GDM patients	Infloran®	4 weeks	↓ FPG, fasting insulin level, HOMA-IR	Glycemic control	[35]
GDM patients	*L. rhamnosus* HN001	—	↓ FPG	Reduced the GDM prevalence	[36]
			↓ Relative rate of GDM		
Pre-diabetic patients	Probiotics (*L. acidophilus, B. lactis, B. bifidum, *and* B. longum*); Synbiotic (probiotics + inulin)	24 weeks	↓ FPG, fasting insulin level, HOMA-IR, HbA1c	Glycemic improvement	[37]
			↑ QUICKI score		
Pre-diabetic patients	Probiotics (*L. acidophilus, B. lactis, B. bifidum, *and* B. longum*); Synbiotic (probiotics + inulin)	24 weeks	↓ Hyperglycemia, hypertension	Improved the metabolic syndrome	[38]
			↓ Metabolic syndrome associated parameters		

T2D: Type 2 diabetes; DKD: Diabetic kidney disease; IL-18: Interleukin-18; GFR: Glomerular filtration rate; HOMA: Homeostasis model of assessment; HOMA-IR: Homeostasis model of assessment-insulin resistance; VLDL: Very low-density lipoprotein LDL: Low-density lipoprotein; HDL: High-density lipoprotein; hs-CRP: High-sensitivity C-reactive protein, GSH: Glutathione; TG: Triglycerides; ALT: Alanine aminotransferase; GDM: Gestational diabetes mellitus; DM: Diabetes mellitus; HbA1c: Hemoglobin A1c; BMI: Body mass index; TC: Total cholesterol; SOD: Superoxide dismutase; GPx: Glutathione peroxidase; TAS: Total antioxidant status; MDA: Malondialdehyde; TNF-*α*: Tumor necrosis factor-*α*; QUICKI: Quantitative insulin sensitivity check index; CHD: Coronary heart disease; HRQL: Health‑related quality of life; FOS: Fructooligosaccharide.
